# Nonobstetrical Acute Abdomen during Pregnancy as a Consequence of Colorectal Carcinoma Perforation: Case Report and Review of the Literature

**DOI:** 10.1155/2019/3682980

**Published:** 2019-06-17

**Authors:** Žana Žegarac, Željko Duić, Sandra Stasenko

**Affiliations:** ^1^Department of Obstetrics and Gynecology, University Hospital Merkur, 10000 Zagreb, Croatia; ^2^School of Medicine, University of Zagreb, Šalata 3 b, 10000 Zagreb, Croatia

## Abstract

Colorectal carcinoma is a rare but potentially fatal disease complicating pregnancy. It occurs most frequently in patients aged 50, although some studies report increasing incidence in women under the age of 40. Diagnosis of colorectal cancer during pregnancy is usually made at an advanced stage due to unspecific symptoms. We will present a case of an acute abdomen during pregnancy due to colorectal carcinoma perforation in a 33-year-old patient in her 26th week of gestation. Because of her abdominal condition, left hemicolectomy with colostomy was performed. Two hours after surgery, the patient gave birth to a male child weighing 910 g with an Apgar score of 2/6. The pathohistological finding indicated adenocarcinoma of the colon in Dukes stage B.

## 1. Introduction

Colorectal carcinoma (CC) is a rare but potentially fatal disease complicating pregnancy. The incidence is highest in patients aged 50 (the range is between 40 and 74), although some studies report that it is increasingly occurring in women under the age of 40 [1]. Since the incidence of CC is increasing in younger women, it also may occur during pregnancy [2]. High risk groups include patients with familial adenomatosis polyposis coli, hereditary nonpolyposis colorectal cancer syndrome, long-standing inflammatory bowel disease, and those with an extensive family history of colon cancer [3]. Those groups account for a small percentage (5-10%) of all cancer cases, but CC is more likely to occur in younger patients [3].

## 2. Case Report

The patient P. A., aged 33, G3P2, was admitted to the hospital in her 22^nd^ week of gestation, immediately after she felt sudden abdominal pain. Her pregnancy had been developing normally, apart from hemorrhoids and a single occurrence of blood coated stool. Her genital findings were normal. Laboratory tests showed signs of anemia (WBC 9.28x10^9^/L, Neutrophils 83%, RBC 3.33x10^9^/L, Hemoglobin 89 g/l, Hematocrit 0.27, PLT 222x10^9^/L, urine analysis normal, CRP 7.7 mg/L). The ultrasound examination revealed no abnormalities. The consulted surgeon ruled out acute abdomen. Stool which was tested for occult bleeding was negative. Repeated laboratory tests showed signs of anemia with a slight CRP increase (WBC 7.77x10^9^/L, Neutrophils 85%, RWC 3.06x10^9^/L, Hemoglobin 80 g/L, Hematocrit 0.24, PLT 220x10^9^/L, Iron serum 5 *μ*mol/L, CRP 14 mg/L, urine insignificant). Internal medicine specialist was consulted. Because of sideropenic anemia, iron supplements were prescribed. In her 26^th^ week of gestation, the patient was admitted again with severe abdominal pain and signs of acute abdomen. The cervix was shortened and the 2 centimeters were dilated. As the CTG showed contractions, tocolytic drug was administered intravenously. Laboratory findings showed a decrease in haemoglobin and an increase in CRP (WBC 8,24x10^9^/L, Neutrophils 90%, RBC 2,83x10^9^/L, Hemoglobin 77 g/L, CRP 122 mg/L).

Due to the fact that lab findings were suggestive of infection, antibiotic treatment was started (cefuroxime and gentamicin). The abdominal ultrasound showed a relatively small quantity of free intraperitoneal liquid. In the lienal flexure area an eccentric thickening of the intestinal wall was visible which indicated a possible expansive infiltrative process. An X-ray of the abdomen was taken which showed a narrow transparency below the right hemidiaphragm, possibly free air from an earlier encapsulated perforation. Acute abdomen caused by diffuse peritonitis as a consequence of a perforation of the descending colon tumor was diagnosed and the patient transferred to surgery where laparotomy, left-side hemicolectomy, and colostomy were performed. Two hours after the operation and in spite of the intravenously administered tocolytic medication the patient gave birth to a male child weighing 910 g with an Apgar score of 2/6. On the eighth day after surgery the patient was discharged after ablactation. Histologically, the placenta as well as the amniotic sac and the umbilical cord displayed no pathological alterations. Histopathology report is as follows: the material is a 30 cm long colon segment. A tumor of 3 cm in diameter is located 3 cm from the aboral resection edge filling the entire circumference of the intestinal wall, infiltrating its entire width and spreading into fat tissue. In the underlying fat there are eight normal-looking lymph nodes 0.3-07 mm in diameter. Adenocarcinoma colonis et Peritonitis acuta suppurativa (Dukes B, Astler-Coller B2, TNM: T3, N0, Mx).

## 3. Discussion

The first case of rectal cancer in a pregnant woman was reported by Cruveilhier in 1842 [4]. The incidence rate of CC is controversial; some studies report an incidence of 0.002% while others consider 0.07-0.1%, which amounts to approximately 1 case per 13000 gestations [2]. The mean age of pregnant women with CC has been reported as 31 years of age, with a range of 16-48 years [5]. CC is among the eight most common malignancies in pregnancy [6]. When discovered during pregnancy 64-86% of CC in gravid women tend to involve the rectum and sigmoid colon, unlike the general population where about 65% arise from the proximal colon [7]. Among the cases described so far there is one involving sigmoid colon perforation during pregnancy due to carcinoma discovered in the 28^th^ week of gestation which manifested by abdominal pain and peritonitis [8]. The pathogenesis of CC is still unknown. Possible causes include the influence of oestrogen and progesterone on the growth of tumor cells, since the majority of CC cases displays an increased amount of oestrogen (20-54%) and progesterone receptors (10-100%) [6]. However, all reports did not support this hypothesis [6]. Rowan T. Chlebowski et al. have demonstrated in a randomized trial that the use of estrogen and progestin in postmenopausal women significantly decreases the prevalence of colorectal carcinoma [9]. The understanding of the role of the hormones in the aetiology of CC is limited and conflicting [2]. The previous research on the role of hormones in the genesis and progression of CC needs clarification in future studies [9].

Discussions about the aetiology of colorectal carcinoma further mention diverse growth factors (IGF-1i VEGF), the pregnant women's immune system, and genetic predisposition [7, 10]. Genetic defect, such as mutation of the tumor suppression gene p53, can play a critical initiating role [7]. The symptoms of CC are nonspecific and include fatigue, abdominal pain, nausea, constipation, vomiting, anemia, and rectal bleeding. Rectal bleeding may be attributed to hemorrhoids, which are common in pregnant women [11]. Laboratory abnormalities, including iron deficiency anemia, occur commonly during pregnancy [12]. The similarities between symptoms of pregnancy and of CC are the main reason for delayed diagnosis [11].

A research including 119 pregnant women with colon carcinoma showed that in 47% of them the most frequent symptom was bleeding; 37,6% suffered from abdominal pain and 14% from obstipation, and in 2,4% a perforation occurred [10]. Several other studies reported that CC can develop during pregnancy without any symptoms [2]. 15-30% of people with CC present with an acute abdomen, such as intestinal perforation, mechanical ileus, or bleeding from the gastrointestinal tract [13]. Colonic perforation causes serious peritonitis with life-threatening sepsis. In such a case emergency surgery is required. Colonic perforation is divided into perforation due to colorectal cancer, and benign colonic disease. The reported incidence of malignant perforation due to colorectal cancer ranges from 1,2 to 9 % [14]. Two types of perforation occur in colorectal cancer: perforation at the site of the tumor due to necrosis, which is the most frequent, and perforation secondary to acute obstruction, which is less frequent [15]. The rates of operative mortality of perforated colorectal cancer patients range from 12 to 43% [16]. Perforation is most commonly diagnosed intraoperatively and the confirmation is based on histopathological examination made during the operation or performed after surgery [17].

CC during pregnancy is diagnosed by ultrasound, X-ray, or MRI of the abdomen [18]. Increased risk of placental abruption as a consequence of mechanical pressure against the uterus makes colonoscopy relatively contraindicated. CT is not recommended either [19].

Surgery is the gold standard. If a patient is diagnosed with colorectal carcinoma in early pregnancy an abortion is advised [7]. Up to the 20^th^ week of pregnancy, surgery may be performed and the pregnancy continued. Beyond that point, the 32^nd^ week can be awaited so that fetal maturity is reached and then a Caesarean section, if indicated, and radical surgery may be performed [6]. Upon vaginal delivery, radical surgery should be performed after several weeks [11].

Pregnant women suffering from colorectal carcinoma are prone to metastases to the ovary with a documented incidence of 25% and they generally have a less favourable prognosis. During surgery both ovaries should be subject to biopsy and bilateral adnexectomy should also be considered [11]. Before the 20^th^ week of pregnancy, ovarian biopsy is not recommended on account of the increased abortion and preterm birth rate [18].

Colon carcinoma during pregnancy has no negative influence on the fetus. Metastases in the placenta were described in only one case of colorectal carcinoma. The main problem is a higher incidence of preterm births [4]. Pregnant women suffering from CC show a higher incidence of puerperal infections, which can be explained by immunosuppression caused by the malignant disease [2]. The prognosis of colon cancer in pregnancy is very poor and it worsens as the pregnancy progresses. Diagnosis can be delayed because of the similarity between the symptoms of pregnancy and of CC.

CC is mostly discovered in Dukes B stage (77%) and in C stage (33%) [7]. CC located at the rectum has better prognosis for being diagnosed earlier [10]. CC of the proximal colon can show no signs until it causes obstruction or perforation with an acute abdomen and has a worse prognosis [10].

## 4. Conclusion

Colorectal cancer during pregnancy is a rare but aggressive malignancy with a poor prognosis. Because presentation can overlap with the signs and symptoms of pregnancy, diagnoses are often a challenge. Diagnosis of colorectal cancer during pregnancy is usually made at an advanced stage due to unspecific symptoms. An interdisciplinary approach between a gynecologist and a general surgeon is required. In our department in the last 23 years at 47,000 births, one case was diagnosed.
